# Virulence Factor Genes Incidence among Enterococci from Sewage Sludge in Eastern Slovakia following Safety Aspect

**DOI:** 10.1155/2019/2735895

**Published:** 2019-10-07

**Authors:** Andrea Lauková, Viola Strompfová, Jana Ščerbová, Monika Pogány Simonová

**Affiliations:** Institute of Animal Physiology, Centre of Biosciences of the Slovak Academy of Sciences, Šoltésovej 4-6, Košice 04 001, Slovakia

## Abstract

The sewage sludges represent a potential health hazard because of the quantity of different microbiota detected in sewages. Among microbiota detected in sewages, also belong representatives of the phylum *Firmicutes*. In the past, environmental enterococci in addition to coliforms were widely used as indicators of faecal contamination. Regarding the enterococcal strains as potential pathogenic bacteria, their pathogenicity is mainly caused by production of virulence factors. Therefore, the aim of the study was to analyse incidence of virulence factors in enterococci from cows' dung water. Species identification of 24 enterococci using MALDI-TOF MS system allotted 23 strains to the species *Enterococcus faecium* with highly probable species identification and *E. faecalis* EEV20 with a score value meaning secure genus identification/probable species identification. Enterococci were absent of cytolysin A gene, hyaluronidase gene, and element IS gene. It can be concluded that they are not invasive which is very important from safety aspect. The most frequently detected gene was adhesin *E. faecium *(*efaAfm*, in 22 *E. faecium* strains and in one *E. faecalis*). Adhesin *efaAfs* gene was detected in *E. faecalis* EEV20 and in two *E. faecium*. *GelE* gene was present in three strains. *E. faecium* EF/EC31 was absent of virulence factor genes.

## 1. Introduction

Stabilized animal sewage sludge, including cow's dung water is frequently used for agricultural purposes to farmland application due to its high organic matter content serving, e.g., as a source of nutrients for plants [1]. However, it has brought the hygienic and/or safety aspect into focus. That is, the sewage sludges represent a potential health hazard. The hazards are mainly associated with the amount of different microbiota detected in sewages, especially pathogenic species [2]. Stiborová et al. [1] assessed sewage sludges in Czech Republic through both taxonomic and phylogenetic approaches. There, the bacterial community dominated was affiliated with *Proteobacteria* including the phyla *Deinococcus-Thermus* and *Thermotogae*. The most frequently detected genera in the sludge in Czech Republic were *Mycobacterium* and *Streptomyces *[1]. However, they also detected the phylum *Firmicutes*. In the framework of the phylum *Firmicutes*, Lauková et al. [3] reported representatives of the genus *Enterococcus*. Previously, environmental enterococci in addition to coliforms were widely used as indicators of faecal contamination [4]. Regarding the enterococcal strains as potential pathogenic bacteria, their pathogenicity is mainly caused by production of virulence factors and/or resistance to antibiotics [5]. Therefore, the aim of this study was to analyse incidence of virulence factors in enterococci isolated from cow's dung water (in Eastern Slovakia), which is useful to know from at least two aspects—characteristic and properties of enterococci from different niches in the framework of the basic research and from safety aspect. That is, in sewage sludge could perform possible conjugative transfer or another way of virulence factor gene transfer among strains which can threaten human population.

## 2. Materials and Methods

### 2.1. Sampling, Strain Management, and Identification

Bacterial strains tested (24) were isolated from cow's dung water (sewage sludge) as previously described by Lauková et al. [3]. They were collected from the basins of 25 cattle farms in 15 North-Eastern Slovakian districts. Forty-five samples were transported in bottles and analysed. They were treated according to ISO (International Organization for Standardization), diluted in Ringers solution (Oxoid), and appropriate dilutions were spread plated onto M-Enterococcus agar (Becton and Dickinson, Cockeysville, USA). Incubation was performed in a CO_2_ atmosphere at 37ºC for 24–48 h. Isolated strains were phenotyped with the API 20 Strep system (API, Biomerieux, L‘Etoile, France). Then, they were stored using a freeze dryer (MicroModulyo,Thermo corp., Asheville, Nebraska, USA). However, before testing of virulence factor genes, strains were re-covered in MRS broth (De Man-Rogosa-Sharpe, Merck, Germany) by cultivating at 37ºC for 24 h following their plating on Brain heart agar enriched with sheep's blood (5%), and then plated on M-Enterococcus agar (Difco, Detroit, Michigan, USA). Pure strains were stored for next analyses with the Microbank system (Pro-Lab Diagnostic, Richmond, Canada).

Besides phenotypization, species identification was performed using matrix–assisted laser desorption ionisation time-of-flight mass spectrometry (MALDI-TOF MS, Bruker Daltonics, Billerica, Maryland, USA); [6], meaning Biotyper^TM^ identification system (Bruker Daltonics, USA [7]). This system is used especially for research microbiology. The method is based on analysis of bacterial proteins (fingerprints) using a Microflex MALDI-TOF mass spectrometer. Briefly, a single colony from M-Enterococcus agar was mixed with the matrix (*α*-cyano-4-hydroxycinnamic acid and trifluoroacetic acid) and the suspension was spotted onto a MALDI plate and ionized with a nitrogen laser (wave-length 337 nm, frequency 20 Hz). Lysates of bacterial cells were prepared according to the producer's instructions (Bruker Daltonics). The results were evaluated using the MALDI Biotyper 3.0 (Bruker Daltonics) identification database. Taxonomic allocation was evaluated on the basis of highly probable species identification-score 2.300–3.000, then secure genus identification/probable species identification (value score 2.000–2.299) and probable genus identification associated with score value of 1.700–1.999. Positive controls were *Enterococcus faecium* CCM4231 [8] and *Enterococcus faecalis* DSM 20478 (Bruker Daltonics database 2008).

### 2.2. Detection of Virulence Factor Genes

Genes for seven virulence factors were screened using PCR amplification with the primers and conditions reported by Kubašová et al. [9]. Genes for the following virulence factors were controlled: *gelE* (gelatinase), *esp* (enterococcal surface protein), *efaAfm* (adhesin *E. faecium*), *efaAfs* (adhesin *E. faealis), cylA (*cytolysin A*), hylEfm *(hyaluronidase*), IS16 *(element IS, Table 1). The PCR products were separated by agarose gel electrophoresis (1.2% w/v, Sigma-Aldrich, Saint Louis, USA) containing 1*µ*l/ml ethidium bromide (Sigma-Aldrich) using 0.5x TAE buffer (Merck, Darmstadt, Germany PCR fragments were visualized by UV light. The positive controls were the strains *E.faecalis *9Tr1 (our strain), *E. faecium* P36 (Dr. Semedo-Lemsaddek, University Lisbon, Portugal) and *E. faecium* UW 9086 provided by Dr. Klare from Robert Koch University, Germany). Briefly, the PCRs were carried out in 25 *µ*l volume, the mix consisted of 1x reaction buffer, 0.2 mmol/l of deoxynucleoside triphosphate, 3 mmol/l MgCl_2_, 1 *µ*mol/l of each primer, 1 U of Taq DNA polymerase, and 1.5 *µ*l of DNA template with the cycling conditions previously reported by Kubašová et al. [9].

## 3. Results and Discussion

Species identification of 24 enterococcal strains, done using MALDI-TOF MS system alloted, 23 strains to the species *E. faecium* and one strain to the species *E. faecalis*. Thirteen strains of 23 *E. faecium* (56.5%) were identified with a score value ranging from 2.300 to 3.000, meaning highly probable species identification. Ten strains (43.5%) were alloted to the species *E.faecium* with a score value ranging from 2.000 to 2.299, indicating secure genus identification/probable species identification. The strain *E. faecalis* EEV20 was alloted taxonomically with a score value ranging from 2.000 to 2.299 (Table 2).

All tested enterococci from sewage sludge were absent of cytolysin A-*cylA* gene,hyaluronidase— *hylEfm* gene and element IS (*IS16*) gene. The most frequently detected virulence factor gene was adhesin *E. faecium (efaAfm)*. This *efaAfm* adhesin gene was present in 22 *E. faecium* strains and even in one *E. faecalis *strain (EEV20, Table 2); altogether in 95.8% strains out of all tested. Only *E. faecium* EF/EC31 strain was absent of *efaAfm* gene. On the other hand, adhesin *efaAfs* gene was detected not only in *E. faecalis* strain EEV20 but also in two *E. faecium* strains EF20 and EF/ED21. *GelE* gene was present in three strains, two *E. faecium* (EFV10 and EF20) as well as in the strain *E*. *faecalis* EEV20 (Table 2). Regarding the strains, only one strain-*E. faecium* EF/EC31 was absent of virulence factor genes. *E. faecium* EFV10 had *gelE* gene and *efaAfm* gene; *E. faecium* EF/ED21 had also two virulence factor genes: *efaAfm* and *efaA*fs. Three genes (*gelE, efaAfm, efaAfs*) were detected in the strains *E. faecium* EF20 and *E. faecalis* EEV20.

Mass spectrometry is an analytical technique in which chemical compounds are ionized into charged molecules and the ratio of their mass to charge (m/z) is measured. The development of electron spray ionization (ESI) and matrix assisted laser desorption ionization (MALDI) in 1980s increased the applicability of MS to large biological molecules like proteins. In both spectrometries, peptides are converted into ions by either addition or loss of one or more than one proton [10]. MALDI-TOF is now considered to be a real alternative for bacterial identification due to the provision of rapid and specific determination analogous to molecular sequencing techniques, with benefit of significant time and cost savings. Comparing previously identified species using phenotypization, *E. faecium* was predominant species [3] as was also confirmed using MALDI-TOF system here. In addition, the species *E. faecalis* was also identified among those strains previously. However, phenotypization also indicated the representatives of the other species such as *E. casseliflavus* and *E. durans* among those strains; this was not confirmed using MALDI-TOF MS but a higher percentage of strains identified belonged to the species *E. faecium*. Our experience confirmed a high identification score using MALDI-TOF MS technology associated with phenotypization not only in species *E. faecium *[11] but also *E. faecalis* [12]. High quality MALDI-TOF mass spectra were also obtained in identification of environmental bacteria [13]. E.g., Cherkaoui et al. [14] compared MALDI-TOF technology with conventional phenotypic identification of clinical bacterial strains and they found high confidence identifications for 680 isolates, of which 674 (99.1%) were correct with phenotypization. So, implementation of MS as an identification strategy would improve its efficacy in further analyses to study additional properties of strains e.g., in the framework of basic research. Only one limitation of MALDI-TOF MS technology is that the spectral database containing peptide mass fingerprints of the type strains has to be upgraded to involve new species [10, 13]. As formerly indicated, potential pathogenicity of enterococci is associated with the presence of virulence factor determinants/genes or virulence factor production. Gene *IS16* is a marker specific for clinical *E. faecium*/*E. faecalis* strains associated with nosocomial infection. None of our environmental enterococci contained *IS16* gene. Similarly, enterococci tested were absent of *hyl* (hyaluronidase) gene. Hyaluronidase acts on hyaluronic acid and increases bacterial invasion [15]; it can play a role in different inflammations in host organism, e.g., ear inflammation. It was described to be a part of a genomic island located on a plasmid and this was shown to be enriched in hospital-associated, polyclonal subpopulation of *E. faecium *strains [16]. Enterococcal surface protein (esp) supports adhesion of bacteria; enterococci tested were also absent of *esp* gene. Similarly, Kubašová et al. [9] described no occurrence or only rare occurrence of those genes in faecal canine enterococci. In addition, faecal rabbits' enterococci from Pannon White breed of rabbits were free of *hylefm* and *IS16 *genes [12]. On the other hand, the most frequently detected determinants were those encoding adhesin *efaAfm* (95.8%) which is again similar as reported in canine enterococci by Kubašová et al. [9]. This is also in association with the most frequently detected species *E. faecium* in our study because adhesin *efaAfm* is typical for *E. faecium* species [17]. Cyl A, the cytolysin activator (bacterial toxin with hemolytic activity against eukaryotic cells encoded by *cylA* gene [18]; it can even induce tissue damage. Also this *cylA *gene was not present in tested enterococci. Although our strains were not tested to form a biofilm, we would be interested in further parameters because these factors not only participate in invasion and colonization of host but may also contribute to biofilm formation [19]. Gelatinase, extracellular metalloprotease is able to hydrolyze gelatin, collagen and hemoglobin, which has contributed to bacterial adherence and biofilm formation [20]. Occurrence of virulence factor determinants can be influenced with a source of tested strain or also with a species; that is, Eaton and Gasson [17] reported that *E. faecium* strains and *E.faecalis *showed significantly different patterns in the incidence of virulence determinants. Abouelnaga et al. [21] found three strains out of 88 from fermented food free from virulence determinants, and 16% strains from unfermented food were free of virulence factor determinants. Because enterococci tested were mostly absent of virulence factor determinants such as *cylA* gene, IS16 element, *hylefm* gene *esp* gene and rare in *efaAfs, gelE *gene detection; it can be concluded that they are not invasive which is very important from safety aspect. It can be supposed that their occurrence in environment did not represent health risk. However, here no antibiotic profile was shown; but resulting from previous studies, they were mostly susceptible to antimicrobials (bacteriocins) [22].

## 4. Conclusion

To conclude from our results, prevalence of the species *E. faecium* was detected in cow's dung water (sewage sludge from samples in Eastern Slovakia) with few species of *E. faecalis*. Detected strains were, however, mostly absent virulence factor determinants. This indicates that they did not present invasive character and pathogenicity in environment and host regarding safety aspect. Of course, other studies are underway. Moreover, this study is also a contribution to the basic knowledge regarding the environmental enterococci.

## Figures and Tables

**Table 1:** Oligonucleotides used for amplification of virulence genes in enterococci isolated form sewage sludge.

Primer	Locus	Sequence (5'-3')	bp
(1) Cytolysin	*cylA*	F:TAGCGAGTTATATCGTTCACTGTA	1282
		R:CTCACCTCTTTGTATTTAAGCATG	
(2) ESP^a^	*esp*	F: TTGCTAATGCTAGTCCACGACC	933
		R: GCGGTCAACACTTGCATTGCCGAA	
(3) Adhesin EE^b^	*efa*Afs	F:GACAGACCCTCACGAATA	705
		R:AGTTCATCATGCTGTAGTA	
(4) Adhesin EF^c^	*efa*Afm	F: AACAGATCCGCATGAATA	735
		R:CATTTCATCATCTGATAGTA	
(5) Gelatinase	*gelE*	F:ACCCCGTATCATTGGTTT	419
		R:ACGCATTGCTTTTCCATC	
(6) Hyaluronidase	*hyl*Efm	F: GAGTAGAGGAATATCTTAGC	661
		R: AGGCTCCAATTCTGT	
(7) Element IS16	*IS16*	F:CATGTTCCACGAACCAGAG	547
		R:TCAAAAAGTGGGCTTGGC	

(1) Semedo et al. (2003), (2) ^a^enterococcal surface protein, Eaton and Gasson (2001), (3) ^b^adhesin *Enterococcus faecalis*, Eaton and Gasson (2001), (4) ^c^adhesin *Enterococcus faecium*, Eaton and Gasson (2001), (5) Eaton and Gasson (2001), (6) Klare et al. (2005), (7) Werner et al. (2011).

**Table 2:** Score value and virulence factor genes detected in enterococci isolated from sewage sludge.

Strains	Score value	Virulence factor genes		
	*—*	g*elE*	*efaAfm*	*efaAfs*
EF/KK1	2.339	−	+	−
EF/EC2	2.280	−	+	−
EF/EE3	2.170	−	+	−
EF3A	2.369	−	+	−
EF/EC5	2.366	−	+	−
EF/EEV6	2.291	−	+	−
EFP7	2.356	−	+	−
EF9	2.418	−	+	−
EFV10	2.299	+	+	−
EF/EE11	2.399	−	+	−
EF20	2.329	+	+	+
EF/EA21	2.310	−	+	−
EF/ED21	2.077	−	+	+
EF/SA25	2.239	−	+	−
EF/EC31	2.349	−	−	−
WF/EC32	2.300	−	+	−
EF/EC45	2.250	−	+	−
EF/EC46	2.112	−	+	−
EF/EC47	2.202	−	+	−
EF/EC48	2.200	−	+	−
EF11697	2.353	−	+	−
EF1421198	2.367	−	+	−
EF34697	2.379	−	+	−
EEV20	2.281	+	+	+

EF-*Enterococcus faecium*; EE-*E. faecalis*; gelE-gelatinase; efaAfm−; efaAfs−, − it means no gene detected; +, it means, gene was detected; score value in MALDI-TOF mass spectrometry results evaluation.

## Data Availability

The data used to support the findings of this study are available from the corresponding author upon request.
